# Functional outcome of the anterior vaginal wall in a pelvic surgery injury rat model after treatment with stem cell-derived progenitors of smooth muscle cells

**DOI:** 10.1186/s13287-024-03900-3

**Published:** 2024-09-11

**Authors:** Yiting Wang, Yan Wen, Kayla Kim, Hugo Wu, Jerry Zhang, Amy D. Dobberfuhl, Bertha Chen

**Affiliations:** 1 Department of Obstetrics and Gynecology, Stanford University, Stanford, USA; 2 Department of Urology, Stanford University, Stanford, CA 94305 USA; 3 Palo Alto, USA

**Keywords:** Progenitors of smooth muscle cell (pSMCs), Induced pluripotent stem cell (iPSCs), Organ bath myography, Recurrent vaginal prolapse, Pelvic organ prolapse

## Abstract

**Background:**

Stem-cell-derived therapy is a promising option for tissue regeneration. Human iPSC-derived progenitors of smooth muscle cells (pSMCs) exhibit limited proliferation and differentiation, which minimizes the risk of tumor formation while restoring smooth muscle cells (SMCs). Up to 29% of women suffer from recurrence of vaginal prolapse after prolapse surgery. Therefore, there is a need for therapies that can restore vaginal function. SMCs contribute to vaginal tone and contractility. We sought to examine whether human pSMCs can restore vaginal function in a rat model.

**Methods:**

Female immunocompromised RNU rats were divided into 5 groups: intact controls (n = 12), VSHAM (surgery + saline injection, n = 35), and three cell-injection groups (surgery + cell injection using pSMCs from three patients, n = 14/cell line). The surgery to induce vaginal injury was analogous to prolapse surgery. Menopause was induced by surgical ovariectomy. The vagina, urethra, bladder were harvested 10 weeks after surgery (5 weeks after cell injection). Organ bath myography was performed to evaluate the contractile function of the vagina, and smooth muscle thickness was examined by tissue immunohistochemistry. Collagen I, collagen III, and elastin mRNA and protein expressions in tissues were assessed.

**Results:**

Vaginal smooth muscle contractions induced by carbachol and KCl in the cell-injection groups were significantly greater than those in the VSHAM group. Collagen I protein expression in the vagina of the cell-injections groups was significantly higher than in the VSHAM group. Vaginal elastin protein expression was similar between the cell-injection and VSHAM groups. In the urethra, gene expression levels of collagen I, III, and elastin were all significantly greater in the cell-injection groups than in the VSHAM group. Collagen I, III, and elastin protein expression of the urethra did not show a consistent trend between cell-injection groups and the VSHAM group.

**Conclusions:**

Human iPSC-derived pSMCs transplantation appears to be associated with improved contractile function of the surgically injured vagina in a rat model. This is accompanied by changes in extracellular protein expression the vagina and urethra. These observations support further efforts in the translation of pSMCs into a treatment for regenerating the surgically injured vagina in women who suffer recurrent prolapse after surgery.

**Supplementary Information:**

The online version contains supplementary material available at 10.1186/s13287-024-03900-3.

## Background

Pelvic organ prolapse (POP) is a debilitating condition characterized by the downward movement of the vagina and/or uterus through the vaginal opening. Other pelvic structures such as the bladder, bowel, and rectum can also descend behind the vagina and uterus resulting in a variety of genito-urinary and bowel symptoms that severely impact quality of life. POP incidence is 40% in women between the ages 45 and 85 and increases with advancing age [1].

The muscles and connective tissues in the pelvic floor support the pelvic organs, maintaining their position and function. The levator ani muscle is the main support of the pelvic floor. The vagina also plays an important role in supporting pelvic organs, with smooth muscle fibers and connective tissue connected to surrounding muscles and attached to the outer and inner surfaces of the levator ani [2]. Factors such as vaginal childbirth, chronic increased abdominal pressure, pelvic surgery, and aging can weaken the support of the pelvic floor through tissue injury/atrophy and cell apoptosis [3]. Smooth muscle has also been implicated in the pathophysiology of POP [4]. The smooth muscle cells in the vagina contribute to vaginal tone and contractility. Histologically, the vaginal wall of women affected with POP is deficient in elastin fibers and smooth muscle cells, resulting in stiffer biomechanical properties than those of unaffected controls [5, 6].

POP treatment options rely mostly on surgical attempts using patient’s own tissues or synthetic meshes to reinforce or support the deficient pelvic structures and the vaginal wall. POP is associated with an 11% lifetime risk of surgical treatment. Despite surgical intervention, 29% of treated women will experience recurrence of prolapse [7, 8]. The anterior vaginal wall is a common site of prolapse recurrence after POP surgery [9]. Recurrent vaginal prolapse is also treated with surgery that has even higher failure rates [10]. Currently, there are no treatments to prevent recurrence after surgery.

Given the limited options for the treatment of recurrent prolapse and their suboptimal results, there is great interest in using stem cells to restore deficient pelvic tissues. Different types of cells, such as mesenchymal stem cells (MSCs), induced pluripotent stem cells (iPSCs), and stem cell-derived progenitor or precursor target cells have been considered. The advantages of iPSC-based therapies include the following: (1) Cells can be autologous and devoid of ethical and immunological concerns, (2) A large number of cells can be expanded for therapeutic dosing, and (3) Homogeneous cell populations can be produced with defined protocols. However, iPSCs have the potential to develop into teratomas in vivo. To minimize this risk, many groups have differentiated these cells into progenitors of specific cell types for clinical translation, such as iPSC-derived progenitors of smooth muscle cells (pSMCs). The transplantation of human iPSC-derived pSMCs into the urethra of a stress urinary incontinence (SUI) rat model restored urethral sphincter function by regenerating smooth muscle cells and inducing native tissue elastin/collagen III remodeling [11]. Given these encouraging results, we hypothesize that pSMCs may also be effective in restoring function in the surgically injured vagina.

Therefore, in this preliminary study, we sought to examine whether iPSC-derived pSMCs injected into the vagina of a surgically-injured-vagina rat model might improve the contractile function of the vagina and modulate extracellular matrix (ECM) proteins, such as elastin and collagen, in the vagina.

## Materials and methods

### Reprogramming of iPSCs and differentiation of pSMCs

Skin biopsies were collected from three female patients (ages 40–70). Dermal fibroblasts were isolated from lower abdominal skin biopsies and cultured in DMEM/F12 media supplemented with human platelet lysate and 2-mercaptoethanol. The purity of the cultured fibroblasts was confirmed by immunofluorescence of the fibroblast cell marker vimentin using mouse anti-vimentin (Sigma-Aldrich, St. Louis, Missouri) and heat shock protein 47 using rabbit anti-heat shock protein 47 (Lis Biotech, San Antonio, Texas). These cells were free of contaminating epithelial cells that were negative for cytokeratin AE1/AE3, an endothelial cell marker (Chemicon International, Temecula, CA).

Dermal fibroblasts were reprogrammed to iPSCs using a modified mRNA/miRNA reprogramming method. mRNAs encoding *OCT4, KLF4, SOX2, c-MYC,* and *LIN28* as well as the miR-302/367 cluster were transduced [12]. iPSCs clones of each patient were isolated. iPSCs were cultured in mTeSR1 basal medium (Stemcell Technologies, Vancouver, Canada) with 5X supplement (Stemcell Technologies) on recombinant vitronectin (Invitrogen, Waltham, Massachusetts). Conversion from fibroblasts to iPSCs was verified using fluorescence-activated cell sorting (FACS). This showed that 94–99% of the cells expressed pluripotent markers (TRA-1-60, TRA-1-81 and SSEA-4). G-banded cell karyotyping was performed by the WiCell Research Institute (Madison, WI) on each iPSC clone. Clones with a normal karyotype were selected for differentiation into pSMCs.

iPSCs at passage 2 (P2) were differentiated into pSMCs using a feeder-free vascular endothelial progenitor protocol previously described [13]. pSMCs were cultured on human placenta-derived collagen IV (Sigma-Aldrich) and expanded in DMEM/F12 HEPES supplemented with 5% fetal bovine serum (FBS). pSMCs at passage 4 (P4) were used for injection. The pSMCs at P4 were characterized by RT-PCR, immunocytochemistry, and FACS with the smooth muscle cell markers, smoothelin and α-SMA. A cell contractility assay, involving carbachol stimulation, was performed on terminally differentiated pSMCs (P6) to evaluate the contractile function of SMCs compared that of primary bladder smooth muscle cells as previously described [11].

### Generation of the surgically-injured-vagina rat model and cell injection into the vagina

Healthy female immunodeficient Rowett Nude rats (RNU, Charles River Laboratories, Hollister, CA) weighing 150–200 g were used to create the model as described in our published study [14]. Animals were maintained at the Stanford University Research Animal Facility in accordance with the guidelines of Stanford University’s Institutional Animal Care and Use Committee. The animals were anesthetized via intraperitoneal injection of ketamine (30–100 mg/kg) and xylazine for the surgery or via inhalation of 1–4% isoflurane for the cell injections. The animals were euthanized with inhalation of carbon dioxide.

The surgically-injured-vagina rat model was created as previously described [14]. There is robust documentation of changes in the vaginal tissues of women with POP compared to those of unaffected controls. These include decreased smooth muscle content and collagen and elastin expression [5, 15–17]. Therefore, the criteria for successful model construction were decreased tissue contractility due to decreased smooth muscle content demonstrated by organ bath myography and collagen/elastin protein expression in vaginal tissues. The incidence of POP is greater in postmenopausal women; therefore, we induced menopause in the model by ovariectomy. Briefly, an abdominal midline incision was made. The adnexal vascular supply was ligated and the ovaries were resected. To expose the anterior vagina, the urethra was circumferentially detached from the anterior vaginal wall and pubic bone by sharp dissection. To mimic the vaginal injury caused by prolapse surgery, two cuts were made in the anterior vaginal muscularis layer without penetrating the vaginal lumen. This area was then clamped with a hemostat for 5 min and the bladder returned to its normal position in the pelvis. The abdominal incision was then closed. The rats were checked daily for 1 week after surgery, and then once a week until euthanasia. The rat cages were housed in the same room and shelf to minimize confounders. In our published studies, the rat model group (rats that underwent ovariectomy and vaginal surgery) demonstrated consistent reductions in contractility and elastin/collagen protein expression of the vagina and bladder compared to the intact controls (rats that did not undergo any surgery) [14].

Cell injection was performed at 5 weeks post-surgery to allow for inflammatory changes to subside. In the VSHAM group, saline was injected into the right and left sides of the anterior vagina wall. In the cell-injection groups, 2 million cells in 200 μl sterile saline were injected (1 million cells/100 μl/side). This cell dose was selected because we observed an effect on the vagina adjacent to the urethra in our prior studies on pSMCs injected into the peri-urethral region in the SUI rat model. The vagina adjacent to the cell-injected urethra showed restoration of smooth muscle cell content in the muscularis with this cell dose [18]. Unfortunately, the injection procedure could not be performed in a randomized fashion because the cells for each group needed to be prepared at the time of injection as cell survival is time sensitive.

The rats were assigned sequentially to five experimental groups as they arrived from Charles River Laboratories. Randomization was not feasible because of the sporadic availability of the RNU rats during the Pandemic. The five experimental groups were as follows: (1) intact controls (control group without any surgical intervention, n = 12). (2) surgically-injured-vaginal model in which rats were injected with saline (VSHAM group, n = 35). (3–5) model injected with pSMCs derived from patients A, B, and C (cell-injection groups A, B, and C, n = 14/group). Patients A and C are in their 40’s and patient B was in her 70’s. Rats were monitored for 5 weeks after injection.

#### Sample size calculation

Based on a pilot study, our primary outcome measure was the vaginal tissue contractile response to KCl stimulation. We estimated the expected effect size to be 0.2 g/cm^2^ (one times the contraction size measured in the VSHAM group) with a standard deviation of 0.2. This gave us a standard effect size of 1.00 which yielded a sample size of 12 rats per group for power of 80% and a two-tailed α of 0.10 [19]. The higher number of rats in the VSHAM group is due to the need of tissues for multiple assay comparisons.

### In vivo bioluminescence imaging (BLI) of transplanted pSMCs

A human luciferase-tagged iPSC line (Huf-5) was used differentiate luciferase-tagged pSMCs to track pSMCs after injection into the vagina. These luciferase-tagged pSMCs were injected bilaterally into the anterior vagina (1 million cells/100 μl saline/side) of control (n = 4) and model (n = 4) rats 5 weeks after surgery. D-luciferin (Biosynth, Itasca, IL) was injected intraperitoneally at 375 mg/kg body weight before image acquisition on day 0, day 2, week 1, week 2, and week 5. Transplanted cell survival in vivo was monitored via bioluminescence imaging with Lago X (Spectral Instruments Imaging, Tucson, AZ). The photons emitted from luciferase-expressing cells were collected with integration times of 2 min.

### Detection of human gene sequence in harvested tissues from the BLI cell tracking study using an ALU-sequence-PCR assay

The bladder, urethra, vagina, and uterus were harvested from the rats used for the BLI cell tracking study after euthanasia on day 2, week 1, week 2, and week 5. Genomic DNA was extracted from tissue homogenates following the manufacturer’s instructions (QIAGEN, Redwood, CA). The amount of human DNA in each sample was quantified by amplification of a human-specific DNA sequence *ALU* (primer sequences are shown in Table 1). The Brilliant SYBR Green PCR method was used to perform PCR using AriaMx (Agilent Technologies, Santa Clara, CA). All PCR reactions were performed in triplicate for fifty cycles. The number of pSMCs per 25 ng of DNA was calculated using a calibration curve with defined numbers of pSMCs.

**Table 1:** Primers used for *Alu*-seq-PCR and RT-qPCR

	Forwoard primer	Reverse primer
human-specific DNA sequence *Alu*	GGTTCAAGCGATTCTCCTGC	GGTGAAACCCCGTCTCTACT
Elastin	ATCGGTGGCTTAGGAGTCTCAACA	TGGAAGACCGACACCAGGAACTTT
Collagen I	GAAGGCAACAGTCGATTCACC	GACTGTCTTGCCCCAAGTTCC
Collagen III	TGATGGGATCCAATGAGGGAGA	GAGTCTCATGGCCTTGCGTGTTT
GAPDH	GCCAGCCTCTCTCATAGACA	TGGTAACCAGGCCGTCCGATA

### Tissue collection and organ bath myography of the five experimental groups

The vagina, bladder dome, trigone and urethra were harvested 10 weeks post-surgery (5 weeks post-injection). Below is a description of the tissue sections used for the different assays.

1. Vagina
  - *Proximal portion* One half was fixed in paraffin and used for H&E, elastin, and h-Caldesmon staining, and the other half was used for RT-qPCR.
  - The middle portion was used for organ bath myography first, followed by ELISA.
  - The distal portion was stored in a -80℃ freezer.
2. Bladder Dome and Trigone
  - The bladder dome and trigone were evaluated with organ bath myography first, followed by histology, RT-qPCR and ELISA.
3. Urethra
  - The proximal portion was with fixed with Tissue-Tek® O. C. T. compound and used for elastin staining.
  - The middle portion was used for RT-qPCR.
  - The distal portion was used for ELISA.

The organ bath myography methodology was described in detail in our previous publication [14]. Briefly, the middle portion of the vagina was mounted circumferentially, and the bladder dome and trigone were mounted longitudinally. The contractile responses were monitored using a custom-made isometric force transducer, and signals were recorded using Lab Chart 7 (AD Instrument, Colorado Springs, Colorado). First, we assessed tissue contraction using potassium chloride (KCl) solution: 40 mM KCl for the vagina and 160 mM KCl for the bladder tissues. After the tissues reverted to their resting tension by washing with Krebs buffer, we assessed the contraction by using carbachol (Sigma-Aldrich, Missouri), a nonselective muscarinic receptor agonist, at increasing concentrations of 0.625 µM, 1.25 µM, 2.5 µM, 5 µM, 10 µM, and 20 µM. The strips were washed again before verifying true carbachol-stimulated response by adding 1 µM atropine, and carbachol (20 μM) 5 min post atropine. Tissues were washed and viability was checked again using the initial KCl concentration. Tissue contractility data were normalized to the tissue area and expressed as the tension per unit of tissue area (g/cm^2^).

### Reverse-transcription quantitative polymerase chain reaction (RT-qPCR)

RNA extraction and RT-qPCR were performed as described in our published manuscript [14]. RT-qPCR was used to evaluate the mRNA expression of elastin, collagen I, and collagen III. The primer sequences are shown in Table 1. RT-qPCR was performed in duplicate on the Aria Mx Real-Time PCR system using Brilliant SYBR Green PCR Master Mix as described previously [11]. GAPDH was used as an endogenous reference.

### Enzyme-linked immunosorbent assay (ELISA)

The protein expression of elastin, collagen I, and III was quantified in duplicate with ELISA kits (Lifespan Biosciences, Seattle, WA) as per the manufacturer’s instructions. Optical absorbance was measured with a SpectraMax M3 spectrophotometer (Molecular Devices, San Jose, CA). The target proteins were quantified based on the standard curve and then normalized to the concentrations of the protein (mg/ml) in the samples.

### Elastin staining and qualitative scoring of elastin in vaginal and urethral tissues

Proximal vaginal tissues were embedded in paraffin and proximal urethral tissues were embedded in OCT. Elastin staining on paraffin and OCT were performed as described previously [20, 21]. Briefly, elastin fibers were stained in Weigert’s resorcin-fuchsin solution (Electron Microscopy Sciences, Hatfield, PA). Cell nuclei were stained with Weigert’s iron hematoxylin working solution (Poly Scientific R&D Corporation, Bay Shore, NY). After washing in running water, the slides were placed in van Gieson’s solution for 3–5 min for collagen fiber staining.

The slides were evaluated semi quantitatively by four observers who were blinded to the study allocations. These observers were instructed to compare and score each morphological criterion to the reference standard, which was a slide prepared using the tissue sections from a rat that did not undergo surgery (intact control) [22]. Compared with those of the reference, the morphological features and scores of the elastin fibers were as follows: fiber length (1= shorter, 2 = intermediate, 3 = similar to the reference), thickness (1 = thinner, 2 = similar to the reference, 3 = thicker), and density (1 = sparser, 2 = similar to the reference, 3 = denser).

### h-Caldesmon (h-CALD) staining and vaginal smooth muscle layer quantification

We performed immunohistochemical staining for h-CALD, a marker for SMC’s, to examine and quantify SMCs. The slides of proximal vaginal tissue were deparaffinized, retrieved and blocked. The sections were incubated with a mouse anti-h-CALD antibody (1:100, Santa Cruz Biotechnology, Santa Cruz, CA) at 4 ℃ overnight and then incubated with a second antibody (horse anti-mouse-biotin, 1:50, Themo Fisher Scientific, Waltham, MA). The ABC kit was used to develop the red color (Vector Laboratories, Burlingame, CA).

Because of the surgical procedures, some parts of the vagina were much thinner than other parts, so we measured the thinner and thicker parts separately. The thinner part of the vagina is likely the surgically injured area. Six measurements were taken on the thinner part and another 6 were taken on the thicker part. Zen software (blue edition, version 3.4, ZEISS, White Plains, NY) was used to measure the thickness. This was performed by two separate people. The average of 12 measurements (from the two examiners) was reported for each part of the vagina (thinner and thicker).

### Data analysis

Statistical analysis was performed using JMP software version 17 (SAS Institute, Inc., Cary, NC). The results are expressed as the mean ± SD. The nonparametric Wilcoxon test was applied for statistical comparisons between groups. All data points, except for the highest value in each group, were included in the analyses. This was because there was high variability in the highest value in each group. Statistical significance was set at *P* < 0.05. The work has been reported in line with the ARRIVE guidelines 2.0

## Results

### Short-term BLI cell tracking study and ALU-seq-PCR after vaginal cell injection in controls and the surgically-injured-vagina rat model.

**Fig. 1:**
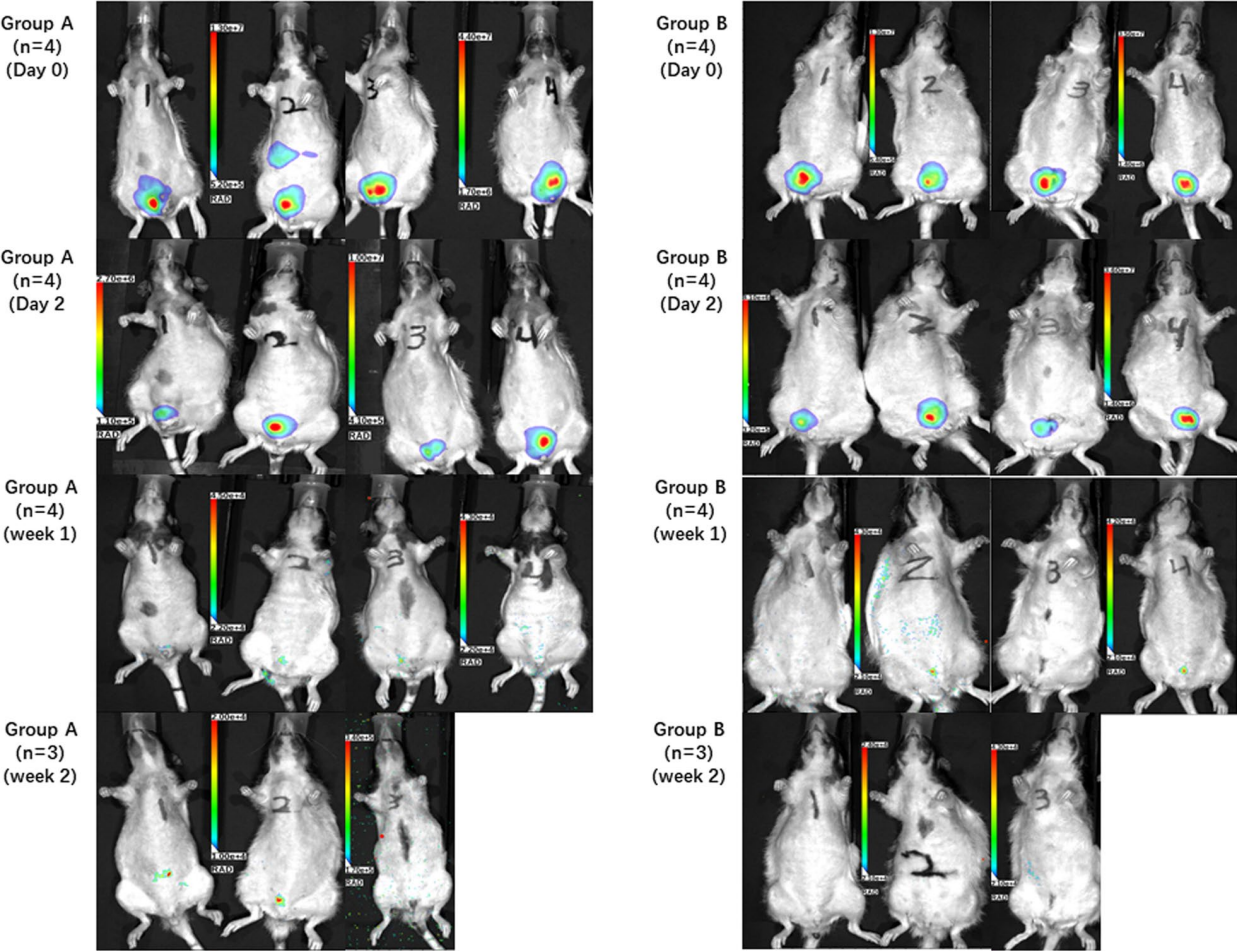
In vivo bioluminescence imaging of transplanted Huf5-luciferase-tagged pSMCs. Group A is the control rats (n = 4), Group B is the model rats (n = 4). BLI signals were detected in all the rats in Groups A and B on day 0 and 2. By week 1, faint signals were detected in some rats from both groups. Faint signals were detected only in Group A by week 2

BLI signals were observed in the pelvis in the area of the bilateral vaginal injection. The signal was faintly present for 1 week in the model group and for 2 weeks in the control group (Fig. 1). Although there was no BLI signal, *ALU*-seq-PCR revealed a calculated total of 900 human cells in the urethra by the end of 5 weeks in the control group. In the model group, no human cells were detected by BLI or *ALU*-seq-PCR after week 1.

### Functional effect of vaginal pSMCs injection on the smooth muscle of the vagina, bladder dome, and trigone

Several rats died due to surgical complications and some of the tissues were damaged due to processing difficulties. Thus, the numbers in each cell-injection group used for the final statistical analysis differed between 10 and 14 rats. The number of saline treated model rats (VSHAM) available for final analysis for each assay also varied depending on which tissue was tested. This is because our preliminary data indicated that we should use the proximal vagina for PCR and histology and the middle vagina for organ bath so only the VSHAM rats that had their vaginas divided this way could be used for comparison (n = 14), whereas all VSHAM rats could be used for the bladder comparisons (n = 35).

#### Vagina

**Fig. 2:**
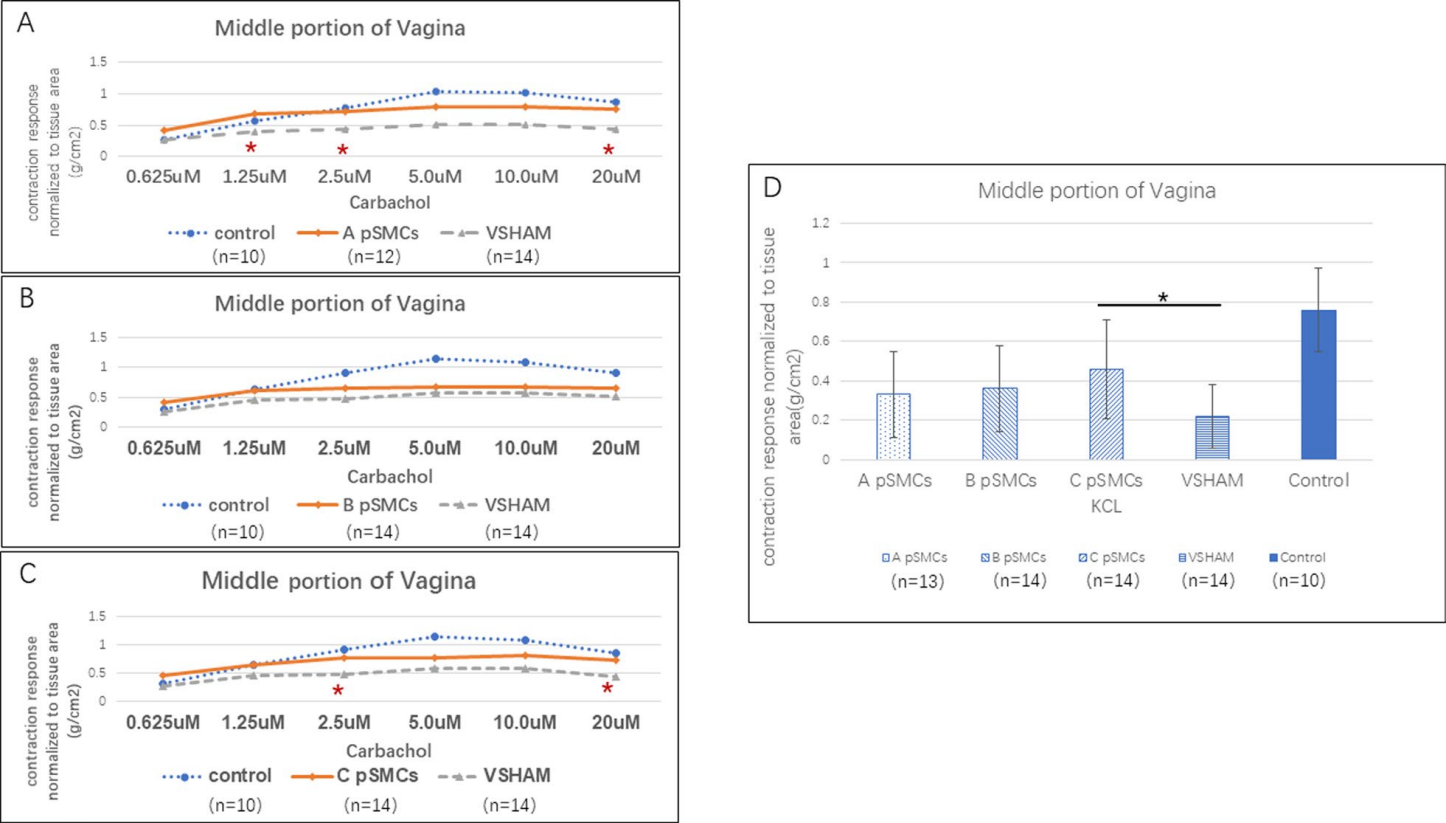
Organ bath myography of the vagina. **A**–**C**. Contraction response of the middle portion of the vagina induced by different concentrations of carbachol, normalized to tissue area, in cell-injection groups (using cells derived from patients A, B and C). **D** contraction response of the vagina induced by KCl in different cell-injection groups. *significant difference compared to VSHAM group, *P* < 0.05

In two of three cell-injection groups (A and C), the contraction response to carbachol stimulation, normalized to the area of the vagina, was significantly greater than that in the VSHAM group at 2.5 µM and 20 µM carbachol concentration (*P* < 0.040). In group A, it was also significantly greater at the 1.25 µM concentration (*P* = 0.0448) (Fig. 2A–C, Table S1). The KCl-induced contraction was significantly greater in the cell-injection group C (*P* = 0.0446) than in the VSHAM group, while cell-injection A and B groups tended to exhibit greater contraction than did the VSHAM group (Fig. 2D, Table S1).

**Fig. 3:**
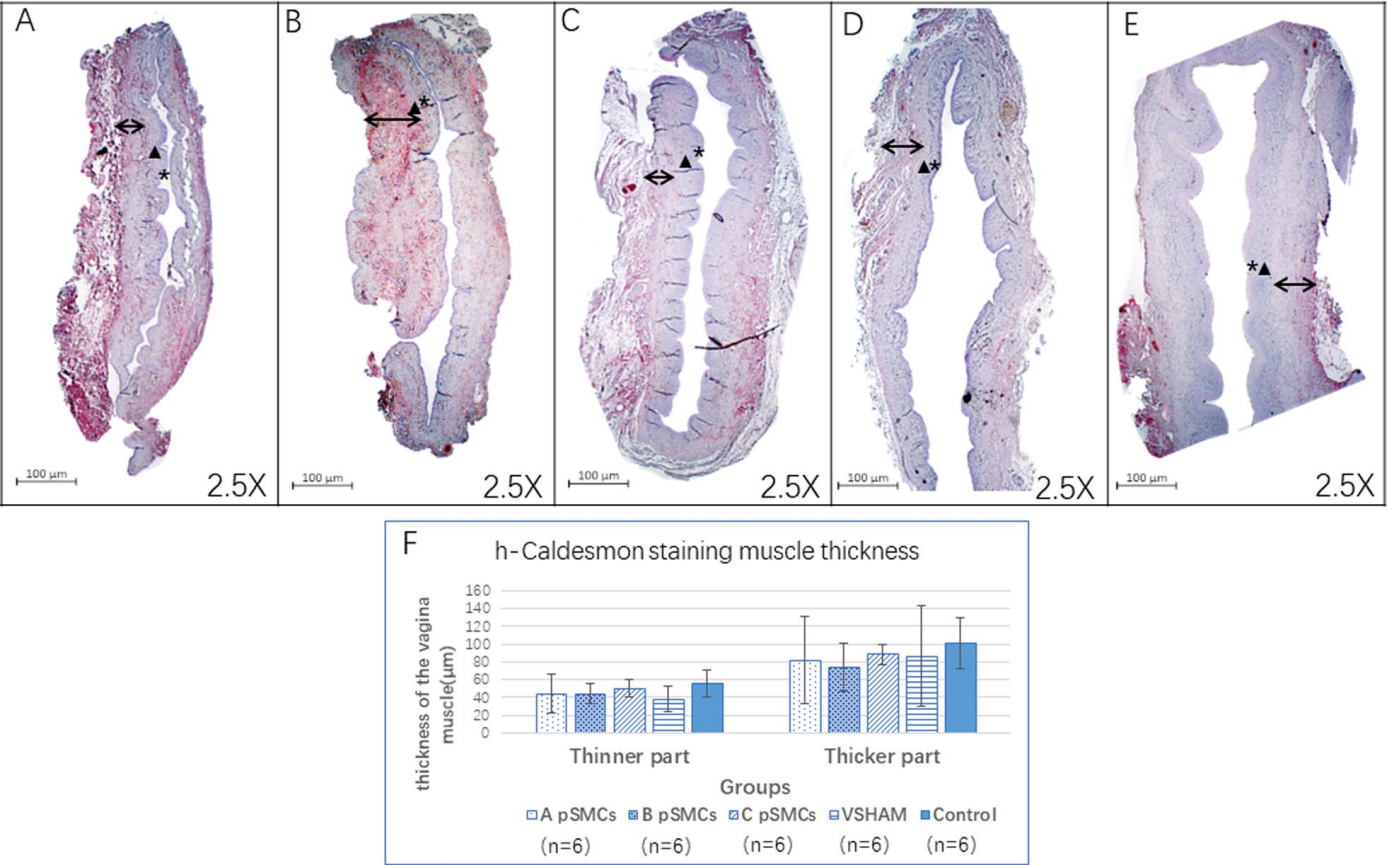
h-Caldesmon staining of the vagina. **A**–**C** cell-injection group ( using cells derived from patients A, B and C). **D** VSHAM group. **E** control group consisting monitored rats that did not undergo any surgery. *: Mucosal layer. ▲: Lamina propria layer.↔: Muscularis layer. **F**. The mean smooth muscle (muscularis) thickness both at the thinner part and thicker part of the vagina. Scale bar = 100 µm

Immunohistochemical staining revealed that the thickness of the thinner part of the vagina in all 3 cell-injection groups tended to be thicker than that of the VSHAM group, although the difference was not significant (Fig. 3. VSHAM: 38.0 ± 14.10 μm, cell-injection group A: 44.4 ± 22.36 μm, *P* = 0.575. Group B: 43.9 ± 11.48 μm, *P* = 0.379. Group C: 49.88 ± 9.68 μm, *P* = 0.065).

#### Bladder dome and trigone

**Fig. 4:**
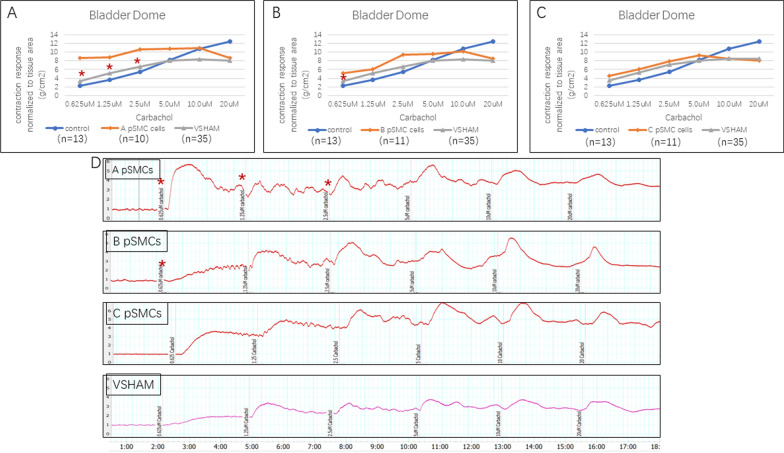
Organ bath myography of the bladder dome. **A**–**C**. Bladder dome contraction response induced by different concentrations of carbachol, normalized to tissue area, in different cell-injection groups (using cells derived from patient A, B and C). *P < 0.05. **D**. Representative organ bath myography tracing of the bladder dome from each cell-injection group at different concentrations of carbachol stimulation

The carbachol-induced contraction response normalized to the area of the bladder dome in the cell-injection groups was significantly greater than that in the VSHAM group at low concentrations (0.625 µM in groups A and B, 1.25 µM and 2.5 µM in group A, *P* < 0.037) (Fig. 4A–C) There was no significant difference in contraction response of the bladder trigone between the VSHAM group and any of the cell-injection groups (data not shown).

### Effect of pSMCs injection on gene and protein expression of collagen I, collagen III, and elastin in the vagina.

**Fig. 5:**
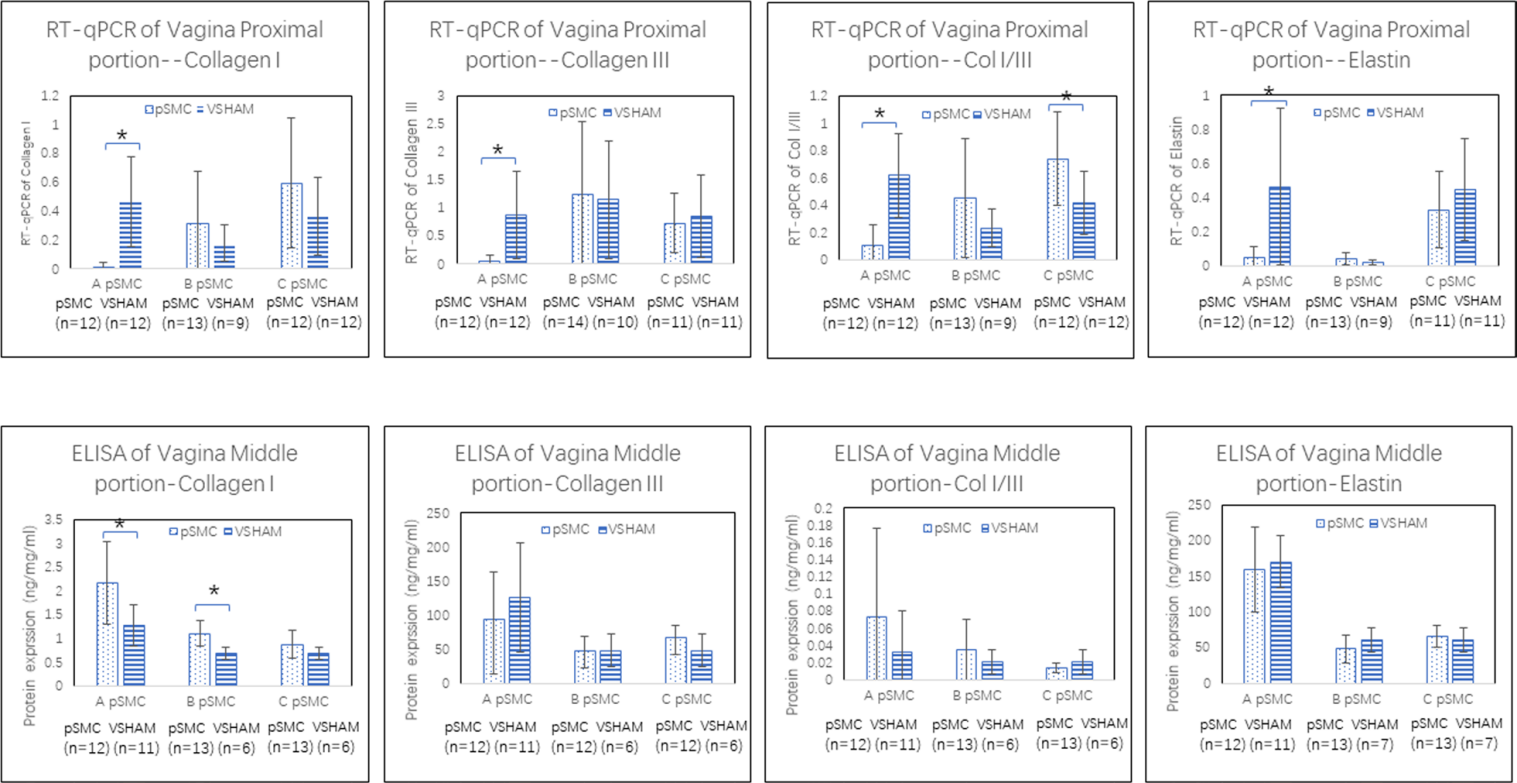
Gene (RT-qPCR) and protein (ELISA) expression of collagen I, collagen III, collagen I/III ratio, and elastin in the vagina of the different cell-injection groups (using cells derived from patients A, B and C) compared to the VSHAM group. *P < 0.05. Error bar = SD

Compared to VSHAM vaginas, collagen I, III, and col I/III mRNA expression in cell-injection groups were not consistently increased or decreased (Fig. 5). However, the protein expression of collagen I in the vagina was consistently greater in the cell-injection groups than in the VSHAM group, with groups A and B being significantly greater (*P* < 0.029). Collagen III protein expression and collagen I/III protein ratio were not significantly different than those of VSHAM.

Neither the mRNA nor protein expression of elastin in the three cell-injection groups showed a consistent trend when compared to that in the VSHAM group (Fig. 5). Moreover, elastin staining scores for both the amount and length of elastin fibers did not differ between the groups (data not shown).

### Effect of pSMCs injection on gene and protein expression of collagen I, collagen III and elastin in the urethra.

**Fig. 6:**
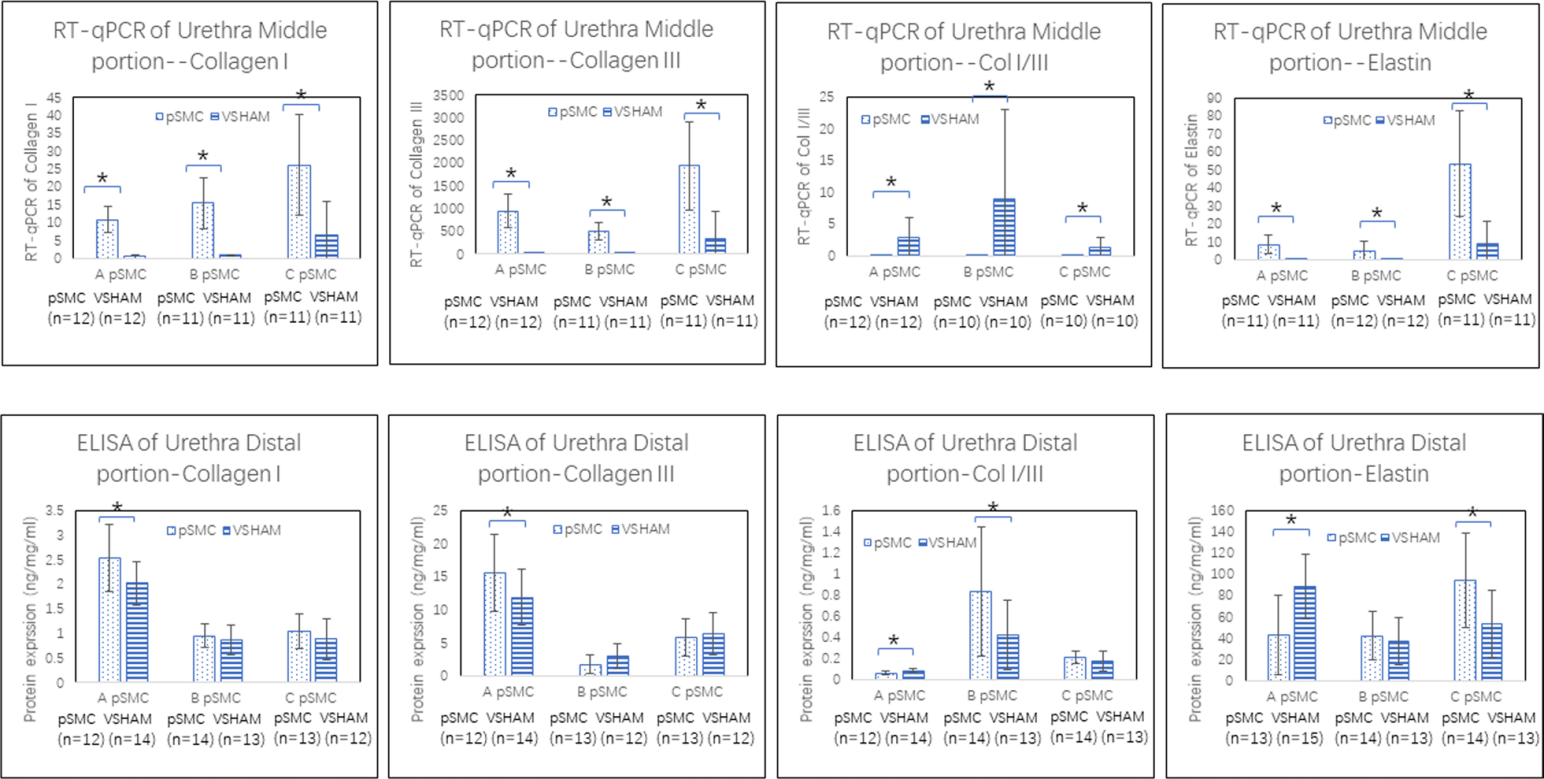
Gene (RT-qPCR) and protein (ELISA) expression of collagen I, collagen III, collagen I/III ratio, and elastin in the urethra in different cell-injection groups (using cells derived from patients A, B, and C) compared to the VSHAM group. *P < 0.05. Error bar = SD

The mRNA expression of collagen I and III in all three cell-injection groups was significantly higher than that in the VSHAM group (*P* < 0.001, Fig. 6). The collagen I/III mRNA expression ratio was also significantly lower for all three cell-injection groups than for the VSHAM group (*P* < 0.0036, Fig. 6), indicating that collagen III expression was higher than that of collagen I.

The protein expression of collagen I in one out of the three cell-injection groups was significantly higher than that in the VSHAM group (*P* = 0.0477) with the remaining two cell-injection groups showing no significant difference. The protein expression of collagen III and the collagen I/III protein ratio was not consistent among the three cell-injection groups (Fig. 6).

Compared to those in the VSHAM group, the urethra of all three cell-injection groups exhibited significantly increased elastin gene expression (*P* < 0.001). No consistent trend was observed in elastin protein expression in any of the three cell-injection groups (Fig. 6).

**Fig. 7:**
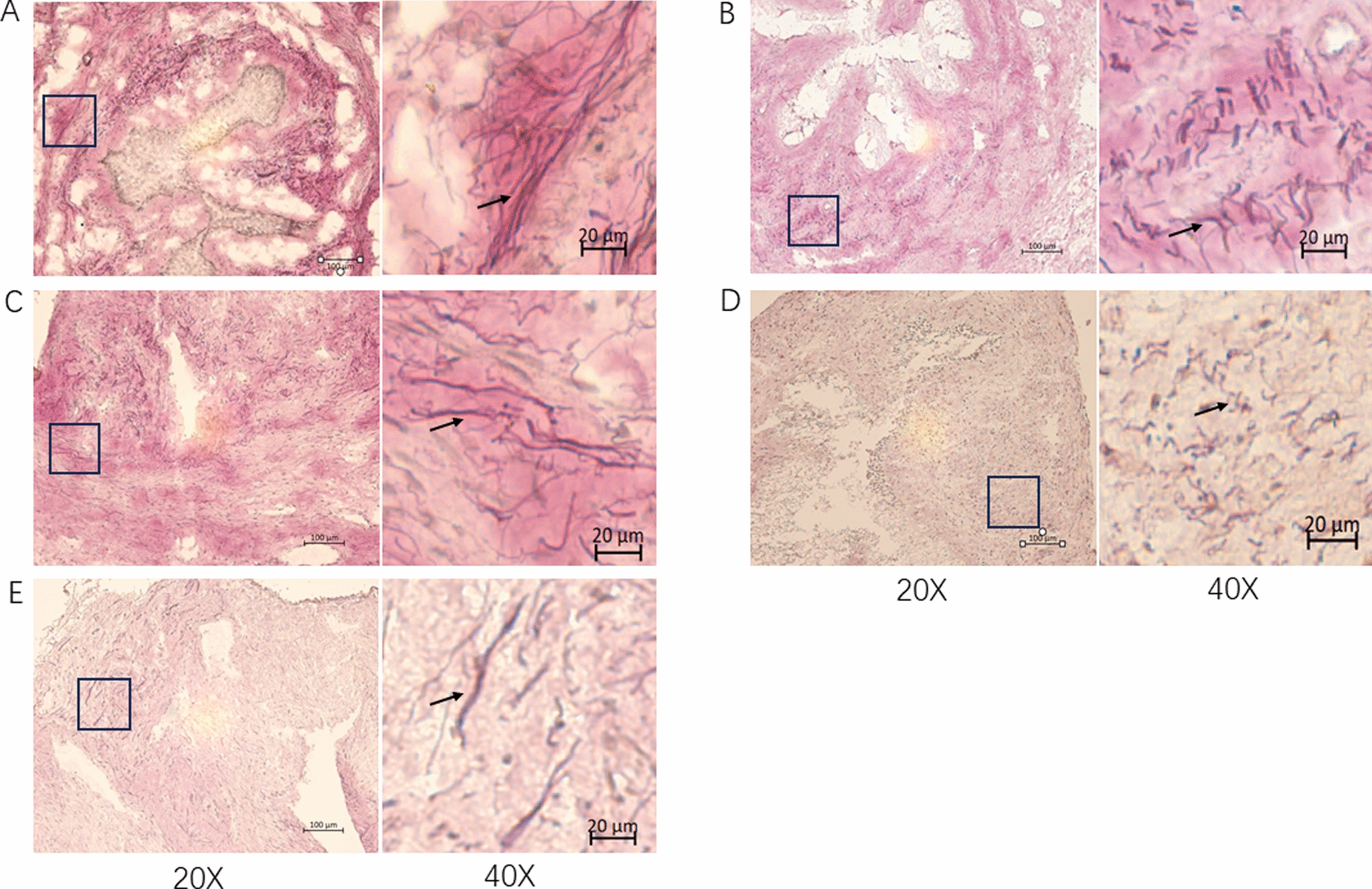
Elastin staining of the urethra. **A**–**C** cell-injection groups (using cells derived from patients A, B, and C). **D** VSHAM group. **E** Intact control group (rats that did not undergo surgery). The arrows indicate the black elastin fibers. Scale bar = 100 µm for ×20 magnification and 20 µm for ×40 magnification

Qualitative analysis of elastin in the urethra (Fig. 7), revealed that the elastin fiber length score in cell-injection group B was significantly higher than that in the VSHAM group (*P* = 0.0074), but the other two cell-injection groups were similar to the VSHAM group. The fiber thickness and density scores were similar for all groups.

### Effect of pSMCs on gene and protein expression of, collagen I, collagen III, and elastin in the bladder

Compared with those in the VSHAM group, the mRNA expression of collagen I and collagen III, and collagen I/III mRNA ratio in the bladder dome did not show a consistent trend in the three cell-injection groups. The gene expression of elastin in the bladder dome was significantly lower in one out of three cell-injection groups than in the VSHAM group (*P* = 0.0005) (Table S2).

Compared with those in the VSHAM group, the mRNA expressions of collagen I (group C, *P* = 0.0035) and III (group B and C, *P* = 0.0134) in the bladder trigone increased significantly with the remaining groups showing an increasing trend. Collagen I/III mRNA ratios were all decreased, with group B showing significance (*P* = 0.0404), compared to that of the VSHAM group. However, the protein expression did not show a consistent trend in the three cell-injection groups.

No significant differences in elastin mRNA expression in the bladder trigone were detected between the cell-injected groups and the VSHAM group. Elastin protein expression in the bladder trigone was significantly lower in two out of three cell-injection groups (groups A and C) than in the VSHAM group (*P* < 0.013) (Table S2).

## Discussion

Smooth muscles play an important role in the function of the vaginal wall [23]. The content of smooth muscle cells (SMCs) in the vagina decreases with age and is less in women with pelvic organ prolapse (POP) than in unaffected women [24–26]. This decrease in smooth muscle content, organization, and composition, which are characteristic of prolapsed vaginal tissue, can negatively affect vaginal tone and contractility [23, 26]. This may be due to the effect of aging on the regenerative ability of muscle by reducing both stem cell pool and functionality [27]. Smooth muscle cells and fibroblasts also contribute to the extracellular matrix of pelvic tissues. Others have also documented differential expression of extracellular matrix proteins in pelvic tissues of women with and without prolapse [28]. Together, these data indicate that several mechanisms contribute to the presence of pelvic prolapse.

Cell therapy, which aims to improve the proliferation or content of SMCs, may be an approach to address SMC loss or decline. There are limited studies using muscle cells to address deficiencies in SMCs. Skeletal muscle-derived cells (SMDCs) injected into the external anal sphincter improved sphincter-related fecal incontinence due to external anal sphincter damage and/or atrophy in both men and women [29]. While bone marrow mesenchymal stem cell-derived smooth muscle cells cultured with rat aortic SMCs in a 3D collagenous milieu showed significant pro-elastogenic and anti-proteolytic effects, increased contractility, and increased elastin production [30]. These publications suggest that SMC-based therapy can not only address cell deficiency but may also modulate the abnormal ECM observed in prolapsed pelvic tissues.

The focus of our group is the translation of human iPSC technology for pelvic floor disorders. Our previous work demonstrated that periurethral injection of human iPSC-derived progenitors of smooth muscle cells (pSMCs) can facilitate the restoration of sphincter SMCs and function in a stress urinary incontinence (SUI) rat model [11]. Given that the anterior vagina is the most common site for prolapse recurrence after POP surgery, in this study, we hypothesized that iPSC-derived pSMCs may have a similar regenerative effect on the surgically injured anterior vagina. Organ bath myography with carbachol and KCl stimulation was used to evaluate the function of the vagina. Carbachol is a cholinergic agonist that mimics the action of the neurotransmitter acetylcholine which induces smooth muscle contraction, and KCl induces smooth muscle contractile function by direct membrane depolarization [31, 32]. Our data showed that both carbachol and KCl induced stronger contractile responses in the cell-injection groups than in the saline-treated group.

We also examined the thickness of smooth muscle in the vagina via immunohistochemistry staining of a SMC marker (h-CALD). We observed that the vaginal thickness in the surgically injured section of the vagina appeared slightly greater in the cell-injection groups than in the VSHAM group, although this difference was not statistically significant. The lack of significance may be due to the high variability of the measurements and the qualitative nature of this assessment modality. Future studies could benefit from a more quantitative method of assessing SMC changes.

Although we did not directly damage the bladder, we separated the bladder and urethra from the uterus and anterior vagina during the model creation. Therefore, we also evaluated the bladder and the urethra in this study. We found that bladder dome contractions in response to carbachol were stronger in most of the cell-injection groups at low concentrations of carbachol and KCl than in the VSHAM group. However, there was no difference in the bladder trigone contractility between the cell-injection and VSHAM groups. The bladder trigone has two muscle layers, an outer layer of detrusor muscle fibers that are parasympathetically innervated and an inner smooth muscle layer that receives noradrenergic sympathetic innervation [33]. We were not able to evaluate nonadrenergic sympathetic innervation due to the length of time that the tissues were exposed to the organ bath with the carbachol and KCl stimulation; therefore, we cannot comment on whether there might be changes in the sympathetic innervation of the bladder trigone.

The extracellular matrix (ECM) provides the substrate on which cells migrate, proliferate, and differentiate [24]. This substrate is dynamic and undergoes constant remodeling in response to external forces; thus, it also modulates the biomechanical properties of the tissue. Collagen I and III are two major protein subtypes in pelvic tissues. Collagen I confers tensile strength, while collagen III confers flexibility and distensibility. Both are present in tissues that are subjected to periodic stress [13, 34].

The ECM in the vagina changes with age, trauma, and conditions such as POP. The literature supports the differential expression of ECM components in women with and without POP; however, the data are not consistent. Some studies have reported an increase in fibroblast, collagen, and elastin fibers with aging, while others have reported poor-quality collagen or elastin fibers in women with POP [24, 26, 35]. Moalli et al. observed an increase in collagen III in the sub-epithelium and muscular layer of the vaginal wall in patients with POP [28]. Chi’s study demonstrated that severe prolapse is associated with imbalanced collagen synthesis, degradation and deposition. The protein content of both collagen I and III in vaginal tissue was reduced [16]. Söderberg investigated the overall collagen content in paraurethral ligaments and found it to be lower in patients with prolapse than in those without prolapse [36].

In the present study, collagen I protein expression was increased in two out of the three cell-injection groups compared to that in the VSHAM group. Collagen III and collagen I/III protein ratio was similar to that in the VSHAM group for all three cell-injection groups. We suspect that this is due to the high variability in each group and to an insufficient cell dose. It is also possible that, because collagen III expression was not significantly altered, the effect of collagen I on tissue biomechanics will be that of increased tissue strength. Because this is a preliminary study, we did not have the ability to evaluate tissue biomechanics. Future studies will include this assessment in order to understand the effect of increased collagen I expression with no significant change in collagen III expression.

Elastic fibers are composed of multiple components, including elastin, fibrillins, and fibulins. These fibers also contribute to the integrity of the vaginal wall by providing extensibility and the ability to recoil with stretching. Hence, intact and mature elastic fibers may also be critical for maintaining smooth muscle cell contractility and vaginal biomechanical integrity [31]. Decreased levels of the elastic fiber components elastin and fibulin-5 in prolapsed human vaginal tissue correlate with POP severity [31, 34, 37]. In the present study, elastin mRNA and protein expression in the vagina was inconsistent between the three cell-injection groups and not significantly different compared to the saline-injection group. These findings suggest that either the improvement in vaginal function after pSMC injection is not directly associated with changes in elastin or that elastin protein expression is not an adequate measurement to describe changes in elastin in the vagina. Some investigators have described morphologic changes in the elastin fibers of the POP vagina [38]. In contrast, the collagen I and elastin mRNA expression in the urethra of the cell-injection groups was significantly greater than that in the urethra of the saline-injection group. These observations of the urethral tissues from the current study are consistent with our previous study in which we observed the same effect of pSMCs on the urethra of the SUI rat model [11]. These data either suggest a differential response to pSMCs between the urethra and the vagina or that the pSMC dose may need further optimization to achieve significant effects in the vagina.

Limitations of our study:

1. *Insufficient sample size* The lack of consistently significant differences is likely due insufficient sample size. Our sample size calculation was based on pilot data suggesting that contractile response to KCl could be used as an outcome measure. However, given the results of this study, we believe the carbachol stimulation is a more reliable outcomes measure. A larger sample size in future studies is recommended to draw appropriate conclusions. We selected two patients in their early 40’s and one in her early 70’s of age to examine if age may affect the results. We did not see a pattern to suggest an age-related effect. The lack of evidence that cells from the oldest participant performed poorly in this study is consistent with our prior data confirming that the iPSC reprogramming process may rejuvenate cells [39, 40].
2. We observed that the injection of pSMC significantly altered ECM gene expression in the urethra, while the results for the vagina were less consistent. In the BLI cell tracking study, cells could not be detected after week 1. Taken together, we believe that a cell dose of 2 × 10^6^ may be insufficient for the vagina. The injected cells are more likely to migrate to the surrounding areas further decreasing the effect on the vagina. Future studies should examine higher cell dosages and incorporate methods to localize the cells to the vagina.
3. Due to the small size of the rat vagina, we needed to use different portions for organ bath myography, PCR, and protein testing. We performed a pilot study (not described in this manuscript) to evaluate the effect of organ bath myography on tissue mRNA expression. Because this pilot study revealed that the organ bath affected RNA expression, we used the proximal vagina for PCR and histology, and the middle vagina for the organ bath and protein evaluation. Nonetheless, the use of different parts for the vagina may have introduced variability.
4. We used h-CALD staining to examine smooth muscle thickness to investigate whether there was an association between the function of the vagina and smooth muscle content after the injection of pSMCs. This is a qualitative, 2D assessment with high variability. This study was limited by vaginal tissue size, future studies would benefit from more quantitative methods for measuring smooth muscle content such as the volumetric density of smooth muscle [24, 41, 42].
5. We were not able to randomize assignment of the rats to the different experimental groups due to Pandemic related rat supply. While the qualitative elastin scores were done with the evaluators who were blinded to the group assignments, all other assays were assessed without blinding. This was done to prevent errors in the assignments as many different assays were performed simultaneously. These factors may have introduced confounders to the results.

## Conclusions

In this preliminary study, we observed that human iPSC-derived pSMCs transplantation appears to be associated with improved contractile function of the surgically injured vagina in a rat model. This is accompanied by changes in collagen I, collagen III and elastin mRNA and protein expressions in the vagina and urethra. These observations support further efforts in translating human iPSC-derived pSMCs into a potential therapeutic option for regenerating the surgically injured vagina in women who suffer recurrent prolapse after surgery.

## Supplementary Information

Supplementary Material 1. Organ bath myography of the bladder trigone. A–C. Bladder trigone contraction response induced by different concentrations of carbachol, normalized to tissue area, in different cell-injection groups. D Representative organ bath myography tracing of the bladder trigone from each cell-injection group at different concentrations of carbachol stimulation.

Supplementary Material 2. Table S1. Contraction response of the middle portion of the vagina induced by carbachol and KCl. Data include mean value, standard deviation and confidence interval.

Supplementary Material 3. Table S2. Gene and protein expression of collagen I, collagen III, collagen I/III ratio, and elastin in the bladder in the different cell-injection groups compared to the VSHAM group. *P＜0.05.

Supplementary Material 4

Supplementary Material 5

Supplementary Material 6

Supplementary Material 7

Supplementary Material 8

Supplementary Material 9

## Data Availability

Data are available upon request. Numerical data on organ bath myography are provided in Supplemental Table S2.

## Abbreviations

iPSCs: Pluripotent stem cells
pSMCs: Progenitors of smooth muscle cells
POP: Pelvic organ prolapse
SUI: Stress urinary incontinence
MSCs: Mesenchymal stem cells
ECM: Extracellular matrix
FBS: Fetal bovine serum
RNU: Rowett Nude rats
VSHAM: Experimental group of surgically-injured-vagina model injected with saline
BLI: Bioluminescence imaging
RT-qPCR: Reverse-transcription quantitative polymerase chain reaction
ELISA: Enzyme-linked immunosorbent assay
OCT: Tissue-Tek® O. C. T. compound
h-CALD: H-Caldesmon
Col I/III: Ratio of collagen I/III
SMDCs: Skeletal muscle-derived cells
