# Supplementary figures and images for: Functional outcome of the anterior vaginal wall in a pelvic surgery injury rat model after treatment with stem cell-derived progenitors of smooth muscle cells
Source: Stem Cell Res Ther. 2024 Sep 11;15:291. doi: 10.1186/s13287-024-03900-3 (PMC11389472; doi:10.1186/s13287-024-03900-3)

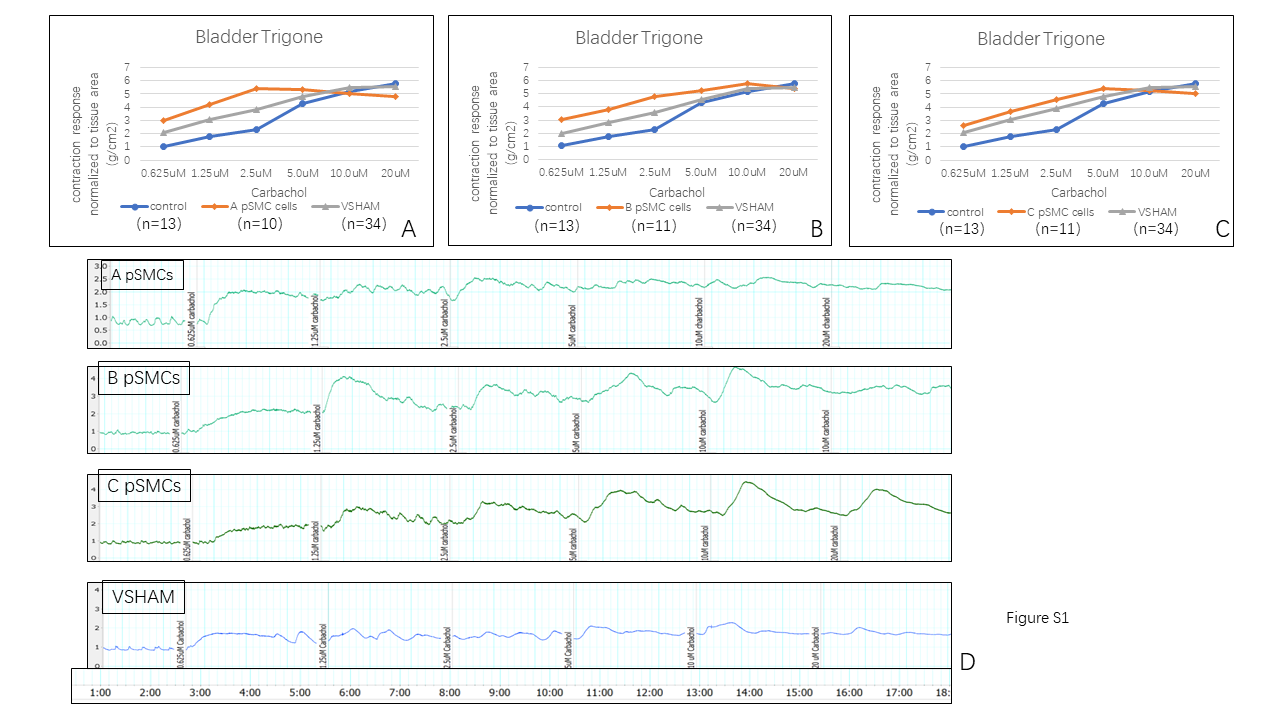

Supplement: Supplementary file 1 — Supplementary Material 1. Organ bath myography of the bladder trigone. A–C. Bladder trigone contraction response induced by different concentrations of carbachol, normalized to tissue area, in different cell-injection groups. D Representative organ bath myography tracing of the bladder trigone from each cell-injection group at different concentrations of carbachol stimulation. [file 13287_2024_3900_MOESM1_ESM.tif]
