# Supplementary material for: Functional outcome of the anterior vaginal wall in a pelvic surgery injury rat model after treatment with stem cell-derived progenitors of smooth muscle cells
Source: Stem Cell Res Ther. 2024 Sep 11;15:291. doi: 10.1186/s13287-024-03900-3 (PMC11389472; doi:10.1186/s13287-024-03900-3)
Supplement: Supplementary file 2 — Supplementary Material 2. Table S1. Contraction response of the middle portion of the vagina induced by carbachol and KCl. Data include mean value, standard deviation and confidence interval. [file 13287_2024_3900_MOESM2_ESM.docx]

Table S1: middle portion of vagina contraction response data supplement, including Mean value, Standard deviation and Confidence interval

Fig 2A-C middle portion of vagina contraction response induced by different concentrations of carbachol, normalized to tissue area

| Vagina middle Carbachol-area | 0.625uM  (mean±SD, 95% CI) | 1.25uM  (mean±SD, 95% CI) | 2.5uM  (mean±SD, 95% CI) | 5.0uM  (mean±SD, 95% CI) | 10.0uM  (mean±SD, 95% CI) | 20uM  (mean±SD, 95% CI) |
| --- | --- | --- | --- | --- | --- | --- |
| Control (n=10) | 0.27±0.25, 0.07 to 0.46 | 0.57±0.38, 0.28 to 0.87 | 0.77±0.43, 0.44 to 1.10 | 1.03±0.37, 0.74 to 1.31 | 1.02±0.34, 0.76 to 1.28 | 0.87±0.29, 0.64 to 1.09 |
| A pSMCs (n=13) | 0.42±0.22, 0.28 to 0.56 | 0.67±0.36, 0.44 to 0.90 | 0.72±0.39, 0.47 to 0.97 | 0.79±0.44, 0.51 to 1.07 | 0.79±0.43, 0.52 to 1.06 | 0.75±0.40, 0.50 to 1.00 |
| VSHAM (n=14) | 0.26±0.25, 0.11 to 0.40 | 0.40±0.28, 0.24 to 0.56 | 0.44±0.24, 0.31 to 0.58 | 0.51±0.35, 0.31 to 0.71 | 0.52±0.38, 0.30 to 0.73 | 0.44±0.29, 0.28 to 0.61 |

| Vagina middle Carbachol-area | 0.625uM  (mean±SD, 95% CI) | 1.25uM  (mean±SD, 95% CI) | 2.5uM  (mean±SD, 95% CI) | 5.0uM  (mean±SD, 95% CI) | 10.0uM  (mean±SD, 95% CI) | 20uM  (mean±SD, 95% CI) |
| --- | --- | --- | --- | --- | --- | --- |
| Control (n=10) | 0.30±0.26, 0.12 to 0.49 | 0.64±0.41, 0.34 to 0.93 | 0.92±0.61, 0.48 to 1.35 | 1.15±0.51, 0.78 to 1.51 | 1.08±0.37, 0.82 to 1.35 | 0.91±0.31, 0.69 to 1.13 |
| B pSMCs (n=14) | 0.41±0.41, 0.17 to 0.65 | 0.61±0.47, 0.33 to 0.88 | 0.66±0.47, 0.39 to 0.93 | 0.67±0.50, 0.38 to 0.96 | 0.67±0.50, 0.11 to 0.40 | 0.65±0.50, 0.36 to 0.94 |
| VSHAM (n=14) | 0.26±0.24, 0.12 to 0.39 | 0.45±0.33, 0.26 to 0.63 | 0.48±0.27, 0.33 to 0.63 | 0.57±0.41, 0.34 to 0.79 | 0.58±0.44, 0.33 to 0.83 | 0.52±0.41, 0.29 to 0.74 |

| Vagina middle Carbachol-area | 0.625uM  (mean±SD, 95% CI) | 1.25uM  (mean±SD, 95% CI) | 2.5uM  (mean±SD, 95% CI) | 5.0uM  (mean±SD, 95% CI) | 10.0uM  (mean±SD, 95% CI) | 20uM  (mean±SD, 95% CI) |
| --- | --- | --- | --- | --- | --- | --- |
| Control (n=10) | 0.30±0.26, 0.12 to 0.49 | 0.64±0.41, 0.34 to 0.93 | 0.92±0.61, 0.48 to 1.35 | 1.15±0.51, 0.78 to 1.51 | 1.08±0.37, 0.82 to 1.35 | 0.86±0.27, 0.65 to 1.07 |
| C pSMCs (n=14) | 0.46±0.34, 0.25 to 0.66 | 0.64±0.35, 0.44 to 0.84 | 0.77±0.35, 0.57 to 0.97 | 0.78±0.22, 0.59 to 0.97 | 0.82±0.31, 0.64 to 1.00 | 0.73±0.23, 0.59 to 0.87 |
| VSHAM (n=14) | 0.26±0.24, 0.12 to 0.39 | 0.45±0.33, 0.26 to 0.63 | 0.48±0.27, 0.33 to 0.63 | 0.57±0.41, 0.34 to 0.79 | 0.58±0.44, 0.33 to 0.83 | 0.44±0.29, 0.28 to 0.61 |

Fig 2D middle portion of vagina contraction response induced by KCl, normalized to tissue area

| Group | Mean value | SD | Lower 95% | Upper 95% |
| --- | --- | --- | --- | --- |
| A pSMCs (n=13) | 0.33 | 0.22 | 0.19 | 0.47 |
| B pSMCs (n=14) | 0.36 | 0.22 | 0.22 | 0.49 |
| C pSMCs (n=14) | 0.46 | 0.25 | 0.59 | 1.16 |
| VSHAM (n=14) | 0.22 | 0.16 | 0.13 | 0.32 |
| Control (n=10) | 0.76 | 0.21 | 0.6 | 0.92 |
