# Supplementary material for: Functional outcome of the anterior vaginal wall in a pelvic surgery injury rat model after treatment with stem cell-derived progenitors of smooth muscle cells
Source: Stem Cell Res Ther. 2024 Sep 11;15:291. doi: 10.1186/s13287-024-03900-3 (PMC11389472; doi:10.1186/s13287-024-03900-3)
Supplement: Supplementary file 3 — Supplementary Material 3. Table S2. Gene and protein expression of collagen I, collagen III, collagen I/III ratio, and elastin in the bladder in the different cell-injection groups compared to the VSHAM group. *P＜0.05. [file 13287_2024_3900_MOESM3_ESM.docx]

| **RT-qPCR of Bladder Trigone and Dome** | | | | |
| --- | --- | --- | --- | --- |
| Organ | **Marker** | **A pSMC (n=11) vs. VSHAM (n=11)** | **B pSMC (n=11) vs. VSHAM (n=11)** | **C pSMC (n=12) vs. VSHAM (n=12)** |
| Bladder Trigone | **Collagen I** | **1.797±1.613 vs. 1.389±0.554 P=0.8438↑** | **1.794±1.055 vs. 1.403±0.536 P=0.4701↑** | **4.680±3.102 vs. 2.138±1.539 P=0.0035↑*** |
|  | **Collagen III** | **3.385±3.137 vs. 2.561±1.473 P=0.7427↑** | **5.266±2.469 vs. 2.855±1.787 P=0.0134↑*** | **7.001±4.285 vs. 3.371±0.733 P=0.0051↑*** |
|  | **Col I/III** | **0.587±0.223 vs. 0.642±0.237 P=0.6650↓** | **0.461±0.170 vs. 0.674±0.254 P=0.0404↓*** | **0.661±0.154 vs. 0.711±0.228 P=0.6236↓** |
|  | **Elastin** | **2.414±2.479 vs. 0.865±0.592 P=0.1486↑** | **0.557±0.294 vs. 0.702±0.483 P=0.5545↓** | **1.519±0.679 vs. 1.115±0.663 P=0.1486↑** |
| Bladder Dome | **Collagen I** | **0.085±0.081 vs. 1.101±0.659 P＜0.0001↓ *** | **1.916±1.514 vs. 1.799±1.726 P=0.6650↑** | **4.882±3.853 vs. 3.594±3.466 P=0.1410↑** |
|  | **Collagen III** | **0.969±1.526 vs. 2.874±2.159 P=0.0039↓ *** | **1.952±0.724 vs. 1.809±1.234 P=0.5205↑** | **8.333±3.405 vs. 7.434±8.098 P=0.2413↑** |
|  | **Col I/III** | **0.215±0.172 vs. 0.443± 0.190 P=0.0014↓ *** | **0.631±0.270 vs. 0.676±0.527 P=0.9770↓** | **0.498±0.215 vs. 0.406±0.294 P=0.1410↑** |
|  | **Elastin** | **0.317±0.137 vs. 1.405±0.779 P=0.0005↓ *** | **0.081±0.047 vs. 0.101±0.148 P=0.4307↓** | **0.155±0.102 vs. 0.223±0.212 P=0.6936↓** |
| **ELISA of Bladder Trigone** | | | | |
| Organ | **Marker** | **A pSMC (n=10) vs. VSHAM (n=15)** | **B pSMC (n=11) vs. VSHAM (n=6)** | **C pSMC (n=11) vs. VSHAM (n=6)** |
| Bladder Trigone | **Collagen I** | **1.010±0.378 vs. 0.704±0.245 P=0.0631↑** | **0.920±0.382 vs. 0.976±0.344 P=0.7131↓** | **0.858±0.176 vs. 0.976±0.344 P=0.8542↓** |
|  | **Collagen III** | **276.9±141.0 vs. 466.4±128.8 P=0.0030↓*** | **421.2±230.2 vs. 451.9±140.1 P=0.7250↓** | **484.2±198.9 vs. 451.9±140.1 P=0.7250↑** |
|  | **Col I/III** | **0.004±0.002 vs. 0.002±0.0003 P=0.0003↑*** | **0.003±0.003 vs. 0.003±0.002 P=0.9599↑** | **0.002±0.0008 vs. 0.003±0.002 P=0.5136↓** |
|  | **Elastin** | **55.37±9.361 vs. 66.16±9.081 P=0.0084↓*** | **44.29±13.16 vs. 58.29±11.52 P=0.0982↓** | **39.53±7.146 vs. 58.29±11.52 P=0.0120↓*** |

**Table S2. Gene (RT-qPCR) and protein (ELISA) expression of collagen I, collagen III, col I/III, and elastin of bladder in different pSMCs groups compared to VSHAM group. *: P＜0.05**
